# Factors influencing the utilization of research findings by health policy-makers in a developing country: the selection of Mali's essential medicines

**DOI:** 10.1186/1478-4505-5-2

**Published:** 2007-03-05

**Authors:** Michael A Albert, Atle Fretheim, Diadié Maïga

**Affiliations:** 1 Department of General Practice and Community Medicine, University of Oslo, P.O. Box 1130, Blindern, N-0318, Oslo, Norway; 2 Norwegian Knowledge Centre for the Health Services, P.O. Box 7004, St. Olavs Plass, N-0130, Oslo, Norway; 3 Direction of Pharmaceuticals and Medicines, Ministry of Health, B.P. E-782, Bamako, Mali

## Abstract

**Background:**

Research findings are increasingly being recognized as an important input in the formation of health policy. There is concern that research findings are not being utilized by health policy-makers to the extent that they could be. The factors influencing the utilization of various types of research by health policy-makers are beginning to emerge in the literature, however there is still little known about these factors in developing countries. The object of this study was to explore these factors by examining the policy-making process for a pharmaceutical policy common in developing countries; an essential medicines list.

**Methods:**

A study of the selection and updating of Mali's national essential medicines list was undertaken using qualitative methods. In-depth semi-structured interviews and a natural group discussion were held with national policy-makers, most specifically members of the national commission that selects and updates the country's list. The resulting text was analyzed using a phenomenological approach. A document analysis was also performed.

**Results:**

Several factors emerged from the textual data that appear to be influencing the utilization of health research findings for these policy-makers. These factors include: access to information, relevance of the research, use of research perceived as a time consuming process, trust in the research, authority of those who presented their view, competency in research methods, priority of research in the policy process, and accountability.

**Conclusion:**

Improving the transfer of research to policy will require effort on the part of researchers, policy-makers, and third parties. This will include: collaboration between researchers and policy-makers, increased production and dissemination of relevant and useful research, and continued and improved technical support from networks and multi-national organizations. Policy-makers from developing countries will then be better equipped to make informed decisions concerning their health policy issues.

## Background

Most health researchers and those who fund health research would like to believe that the work they produce and support is influencing practice and policy and consequently leading to actual improvements in health care delivery. The study of research's influence on policy has had a long and rich background, from early work on the utilization of social science knowledge in government and public policy [1,2], to more recent inquiries into the utilization of systematic reviews by policy-makers [3]. While the various models of policy-processes that have emerged in this field demonstrate that there are many ways in which research may be influencing policy [4,5], it is widely recognized that the level of research utilization by policy-makers is lower than it could be [6]. The body of literature examining the factors influencing the utilization of research findings by policy-makers is increasing. From the two systematic reviews on the subject, common factors are emerging such as: interactions and personal contact between researchers and policy-makers, timeliness and relevance of the research findings, the inclusion of summaries with clear recommendations, mistrust between researchers and policy-makers, and power and budget struggles [3,7]. Most of these findings are however based on studies from industrialized countries, and thus more research is needed into the factors that affect the linkage between research and policy in developing countries. With their limited resources these countries have much to gain from well-informed health policies [8].

The present research focused on one specific health policy: Mali's national essential medicines list (EML). In 1975 the World Health Organization (WHO) introduced the global concept of essential medicines with the first model EML introduced two years later. Updates every two years have lead to the current 14^th ^model list [9]. This reference document is usually used as a starting point for a national list, however each country requires additional information from monitoring and research for its specific health situation [10]. Like many developing countries, the West African country of Mali has a national EML. The Malian list does contain several medicines not present on the WHO model list, including an extra section for "improved traditional medicines." The country's official criteria for the selection of medicines for the list at the time of this study included: harmlessness, efficacy, relevance to the disease pattern, availability on the international market, and cost-effectiveness [11]. Since research findings have much to contribute to these criteria, the factors influencing their utilization, as perceived by policy-makers, were examined.

## Methods

A qualitative approach was used for the present research. By focusing on a specific health policy, participants had the opportunity to concretely discuss their experiences in the policy-making process, including both group and individual experiences. After presenting the protocol to the National Institute of Public Health Research in Mali, the principle investigator was informed that written approval from the ethical committee was not necessary.

### Participants

A purposive sampling technique was used by selecting key informants from the national commission that selects and updates Mali's national EML. The commission is composed of various Ministry of Health staff including health program managers, technical advisors, pharmacists, local medical practitioners considered experts in their field, and technical advisors from the WHO and the European Union. At least one individual from each of these groups was purposively included in the study. One health manager who was not on the commission was mentioned in initial interviews as having played a significant role in the policy-making process, and was recommended for participation. This person was also included in the study. One member of the commission who was available chose not to participate since the interviewer did not have a signed letter from the Ministry of Health. A total of nineteen policy-makers (17 men and 2 women) took part. While there were 33 members on the commission, data saturation had been reached as consecutive interviews yielded diminishing returns of new information and so further interviewing was deemed unnecessary [12].

### Data collection

The principle investigator (MA) conducted 19 in-depth, semi-structured interviews in French (33–89 minutes), between September and December 2005. The interview guide (see Additional file 1: Interview guide) was largely based on the draft interview schedule for assessing research utilization in policy-making provided by Hanney and colleagues as an additional file in their review of research utilization [6]. The topics discussed included: key informants and policy-makers in the policy-process, perceived importance of research findings in the policy-making process, forms of communication found to be useful, different ways research can be used, how well equipped the commission was in absorbing research findings, specific aspects of research that made it useful, presentation/format of research findings, the inclusion of traditional medicines on the list, barriers and facilitators to research utilization, and policy-makers' recommendations of how to increase their utilization of research findings. Participants were asked about specific decisions that may have been difficult to make, including discrepancies between the WHO and Malian EMLs. When "personal expertise" was mentioned as a main source of information in the policy-making process, the acquisition of this expertise through possible utilization of research findings within the individual's career was also discussed.

A natural group discussion, defined as a group interview with "people who know each other already" [12] was also conducted (55 minutes). All 19 interviewees were invited knowing that many would not attend. Four individuals participated in the group discussion. MA gave a short presentation of the research and discussed the preliminary findings. Participants were asked to comment and a group discussion of key topics followed, with MA and DM facilitating.

A document analysis was also performed with the main purpose of validating the information received during interviews and the group discussion. Documents were obtained from the secretary for the commission and the individual who had prepared the technical notes for the commission. The documents covered several updates of the EML that have taken place over the last 10 years. They included the technical notes used by the commission, the documents used to prepare these notes, minutes from meetings, a critical analysis of the country's EML, a synthesis of the decisions made, copies of emails sent to and received from contacts abroad, and Internet printouts.

All but four of the interviews and the natural group discussion were recorded and transcribed by MA, who also took in-depth notes throughout each session. For those that were not recorded – at the requests of the interviewees – MA went over the in-depth notes and produced a detailed summary the same day as the session.

### Analysis

All interviews were analyzed based on Giorgi's phenomenological approach [13]. The analysis followed the following steps: (i) going over all the textual data to gain an overall impression, by MA; (ii) identifying all comments that appeared significant to the research, extracting these meaning units and translating them into English, by MA; (iii) independent abstracting of the meaning units by MA and AF, followed by discussion and consensus; (iv) independent categorization and summarization of abstractions into factors influencing the utilization of research findings as perceived by policy-makers, by MA and AF, followed by discussion and consensus; and finally (v) returning to the extracted text to ensure a good fit with the final factors, by MA. The analysis of the group discussion followed a similar process, however AF only participated in step (v). The document analysis was used to validate statements made in the interviews. Documents were also analyzed to determine the source of the information provided to the commission and to determine the extent to which the information included research findings. Throughout the analysis process preconceptions were shared and critically reflected on between the investigators.

## Results

The factors that emerged from the interviews and group discussion are presented here. The embedded quotations were translated from French by MA and are included here for illustrative purposes.

### Access to information

"The big ticket, at least in the case of Mali, and certainly the case for most African countries where we have problems with quality human resources and regular access to scientific information is the means to allow these people to keep themselves permanently informed," (participant 13, male).

Access to information was discussed in great depth, often accompanied by the statement "we do not have the means". While the Internet and various online resources were mentioned as useful in improving access to research, many of the policy-makers are still not connected and those who are have limited, if any, access to paid sites. Even at the local level it was perceived that the transfer of information from research institutions to policy-makers is poor.

Limited capacity in accessing research findings was also stated as hindering its use. If a policy-maker has extra staff or is a supervisor of students who can search, gather, and compile the information, research findings are more likely to get used. The availability of key texts that supply such information was also seen as facilitating the utilization of research findings. In addition, the more contacts available to policy-makers – be they local experts or international organizations abroad – the greater access policy-makers felt they had to research findings.

Language was discussed as well. Mali's official language is French and while policy-makers agreed that *"the scientific language is very readable," (participant 11, female)*, and could largely be understood even without a solid grasp of English, many also stated that this was a problem. "*It is a serious handicap, serious," (participant 2, male)*. Policy-makers depend greatly on information available to them in French, and this was felt to be limiting. *"If I understood English... I am sure that I could do so much more for everyone," (participant 8, male)*. Additionally, it was apparent from the document analysis that almost all documents used by the commission came from French sources.

### Relevance of research findings

It was stated in the group discussion that most research that is produced is irrelevant to policy-making. Researchers were described as *"doing activities to survive," (participant 7, male)*, and not necessarily to answer questions that need answering. *"That which is pertinent is not funded," (participant 9, male)*. It was proposed that collaboration between researchers and policy-makers could allow policy-makers to give some input into the research process.

### Time consuming process

When discussing the presentation of research findings, interviewees indicated that a lengthy report or publication would not be read, and recommended that researchers provide short and concise documents. In fact, policy-makers stated that research utilization is already a lengthy and time-consuming process. *"Usually I do not have the time," (participant 18, male)*. Even if research is considered important, it still requires a significant amount of time to search, locate, access and review the relevant literature. *"It demands sacrifice" (participant 11, female).*

### Trust in the research

Policy-makers want information they can trust. Those who were able to commission research found this to be important in allowing them to utilize the findings. This was not only because it improved the relevance of the research, but also because it increased the extent to which policy-makers trusted the research. As one policy-maker metaphorically stated: *"If I told you, behind that door there is a cup of tea with sugar and then I give you a cup of tea with sugar here. Which tea can you appreciate? What is outside is not bad, but what is in my hand, that, I can defend," (participant 8, male).*

While no particular form of presentation was stated as being more useful than another, policy-makers indicated that reports should be short (discussed above), and that they would like sections dedicated to methodologies and references. These are considered necessary for determining the quality of the research and therefore whether or not it can be trusted. Policy-makers indicated that their confidence in journals that publish research findings is also relevant. "*If it's in the Lancet or the New England Journal of Medicine, or Science, well, right away we jump on it," (participant 16, male)*. Similarly, research that is supplied by a trusted international organization is more likely to get used. *"If [the research] comes from the WHO, we know it isn't just taken from anywhere... we have confidence in the source," (participant 10, male).*

### Authority of those who present their view

Some interviewees recognized that there is often an uncritical reliance on specialists. Comments made by respected individuals or those deemed extremely knowledgeable in the subject area are highly influential. *"We are often not critical. As a decision-maker we should be going into greater depth... a specialist comes in and we simply say, we're listening... and we write it down. There is no way to contradict, or at least construct contradictory information vis-à-vis the specialist," (participant 13, male)*. This could result in either a decrease or an increase in research utilization. For example, it was stated that a respected professor was useful as leverage in promoting research findings to other members of the commission. At the same time, respected individuals might not be basing their recommendations on research. One policy-maker (participant 7, male) stated that cultural factors might be at play here; as a "*verbal society*" many policy-makers prefer verbal reports to documentation, and place more importance on information that is provided to them by individuals who are highly respected, than documents from a removed source.

### Competency in research methods

Some policy-makers were involved in research studies or programs. Having researchers act as policy-makers was seen as a facilitator to research utilization, and not only because they can provide research findings or act as leverage, but also because they are able to provide a *"veritable training in research methods" (participant 13, male)*. Such training was considered important in improving other policy-makers' competencies in research methods and their ability to understand research presented. While most felt that the commission members were highly qualified, several agreed that more training in research methods would make it easier and further motivate them to utilize research findings. *"There are always nuances in scientific research findings... in order to adapt research findings to make an applicable decision, and certainly when it comes to medicines, I believe the commission members must have a level of qualification sufficiently high in order to effectively exploit the conclusions," (participant 13, male)*. However, one policy-maker who participated in such a training session, claimed to have gained little from the experience. Still others stated that if training is to be provided, incentives to continue working as a low-salaried civil servant must also be included. It was also brought up that increased competency in research methods would not only improve policy-makers' ability to understand the research, but it would also increase the importance they place on research and their motivation to use it.

### Priority/importance of research in making the policy

When asked about the importance of research findings in the policy-making process, policy-makers all stated that, while research is important, other information sources often take precedence. Influence on the policy from higher and lower levels in the healthcare system, such as political will or interests and demands from patients and healthcare professionals tend to be prioritized over research findings.

On the other hand, several policy-makers discussed the fact that this particular policy is highly technical and they could not understand how one could not use research in the decision-making process. *"This is above all else a technical job, scientific. Its basis is science," (participant 3, male)*. The extent to which policy-makers value research findings in the policy process will influence how much it is utilized.

### Accountability

There was some confusion over whose role it is to look up information. Medical professionals and specialists on the commission felt that the technicians in the Ministry of Health were in the best position to access and compile research findings for consideration, while some of these individuals felt that specialists should be supplying relevant findings from their field. Having a specific person or group of persons delegated to search and compile relevant research findings for the policy question at hand was perceived to be extremely helpful. "*You cannot place the responsibility on each person. If you do that it is not going to get done. You have to have a specific group whose job it is to get the information," (participant 13, male).*

## Discussion

By analyzing the selection and updating of Mali's EML, this study discovered several factors that influence the utilization of research findings by health policy-makers in a developing country. Like most studies in this field, this research used qualitative methods with the majority of the data coming from in-depth interviews [6,7,14]. Qualitative methods were chosen as they are "ideal for questions that require an answer about understanding participants' views" [12]. In using such methods, concerns about the validity of the results can arise. It is important to recognize that this study did not measure policy-makers' actual behavior, nor did it measure objective factors that influence their utilization of research findings. Instead, the findings represent policy-makers' *perceptions *of these factors. In addition, this study did not take into account researchers' perceptions of these issues, as has been explored elsewhere [15-19].

By using three separate data acquisition methods, the validity of the findings was improved [20]. In addition to supplementing the information from the in-depth interviews, the natural group discussion provided an opportunity to give feedback to participants, allowing for respondent validation [12]. The selection and number of group members is important for effective group discussions [21], but was a limitation in this study. The results of the document analysis helped to validate participants' statements regarding sources and documents that were accessed. This analysis is limited however because it not known how much consideration, if any, such documents were allotted. The reliability of the results was improved through the use of independent investigators in several stages of the data analysis [22]. There are limitations in the extent to which we can generalize the findings of this study to other policy-makers and other developing countries, due to the exploratory nature of this study and the fact that it examined only one aspect of policy-making in one particular setting.

Two factors emerged from this study that are unique in the literature: the authority of those who present their view and the issue of accountability. These issues may be due to the specific nature of these policy-makers or their cultural setting, and so it is unclear to what extent these factors would be important in other settings. Several of the factors emerging from this study are common in the literature, including: relevance of research findings, trust in the research, and competency or skills of the policy-makers [3,7]. Training policy-makers in research methods and sensitizing them to the usefulness of research findings in the policy process has been recommended in previous studies [18,23,24]. Training policy-makers to develop competencies in acquiring high-quality relevant research and adapting it for local applicability might also be beneficial, since access to information was an important factor in this study and also emerged as a barrier to research utilization in a recent study involving four developing countries – two of which were African [15]. This study also indicated that the lack of value policy-makers placed on research findings was inhibiting its uptake. These negative attitudes towards research have also emerged as a barrier to research utilization in a study conducted in Mexico by Trostle and colleagues [18]. The present study seems to support this finding, and at the same time indicates that if research is considered important in the policy process it may also act as a facilitator to its utilization.

The presentation format of research findings has been mentioned in the literature as a potential facilitator of research utilization, for example by including summaries with clear recommendations [18]. In developed countries, this has been studied further, analyzing whether a specific format such as the "1:3:25" format (1-page take-home messages; 3-page executive summary; 25-page report) would facilitate the utilization of research findings [3]. Policy-makers in the present study did not demonstrate a strong preference for a specific reporting format. The general consensus was that the report should be short, while supplying enough information to allow them to evaluate the quality of the research (by including the methodology and references).

The relative importance of research findings in the policy-process is a complex issue. For example: Mali is one of the few countries in Africa with traditional medicines on its EML. In discussing one of these 'improved traditional medicines," one policy-maker mentioned having knowledge of, and considering, an apparently high-quality study that had concluded that the medicine in question, while somewhat effective, was not the most effective. For this policy-maker, political will and community values were more important than the difference in relative effectiveness demonstrated by the research findings. While the authors might not necessarily agree with the decision made, there is potentially nothing wrong with policies that do not directly follow research recommendations. According to the conceptual approach to the definition of "research utilization" from Nutley and colleagues' adapted from Weiss, research that affects policy-makers understanding of a situation – even if the final decision does not follow directly from researchers' recommendations – is still being utilized [25]. Other influences such as local values and needs are recognized as important inputs into policy [4]. It is, after all, policy-makers who make policy, not researchers. In this example then, even though the research findings were considered less important, they could still be considered as having been used in the policy process. If several factors are working together however, the utilization of research is likely not to occur at all. If using research findings does not take precedence and is already perceived as a time-consuming task in a country where capacity to access this information is low, and no one is quite sure who is responsible to get the information, then chances are the research will not be read or considered, let alone influence the decisions of the policy-maker.

Policy-makers' belief that searching, accessing and reviewing research findings is highly time consuming is perhaps a good argument for the increased production, promotion and dissemination of systematic reviews. Systematic reviews are increasingly recognized as offering many advantages to the target audience [26], including the fact that they lead to a more efficient use of time [3]. No policy-makers mentioned having utilized information from systematic reviews, and most seemed unaware of their existence. Organizations such as the Cochrane Collaboration and the National Institute for Health and Clinical Excellence of the UK are currently leading the way in providing systematic reviews for medical practice. Studies have begun looking at how to improve their usefulness for health care managers and policy makers [3]. Interestingly, the Essential Medicines Department of the WHO actually re-introduced amodiaquine in the treatment of malaria following the publication of a systematic review from the Cochrane Collaboration indicating its safety and effectiveness compared with chloroquine [27]. Much work is still needed before systematic reviews are utilized in common practice in the formulation of policy at national levels, especially in developing countries.

Policy-makers in the present study stated that it was extremely helpful having trusted networks such as multi-national organizations supply information relevant to their decision-making. The fact that the WHO initiated the EML policy and supplied the commission with a model list was felt by one policy-maker to be the main reason why most of the medicines on the national EML were chosen based on evidence. These multi-national organizations can play an important role as knowledge brokers in helping policy-makers in developing countries make informed-decisions through continued and increased technical support, such as the Health InterNetwork Access to Research Initiative (HINARI) set up by the WHO [28-30].

Perhaps the most prevalent theory discussed in the literature on this topic is the "two-communities theory" developed by Caplan and colleagues. It highlights the fact that health researchers and policy-makers have two competing world views [2]. Increased collaboration and personal contact between researchers and policy-makers have been proposed and studied as solutions to the problems related this issue [31]. While these concepts were discussed in the interviews, the authors agreed that they did not emerge as factors *per se*. They were seen as indirect influences. Increased collaboration and personal contact between researchers and policy-makers could lead to increased access to information, improve policy-makers' trust in the research, enhance their understanding of, and competency in, research methods and the skills required to access research findings, and allow them to influence the research and make it more relevant to their own needs. This last point, while seemingly good for policy-makers, can pose potential problems to the objectivity and quality of research produced [7,32].

With the limited number of studies from developing countries on this topic, further research seems necessary. The factors emerging from this study, including those that are not common in the literature, warrant further investigation and should be considered in the planning of strategies to bridge the gap between research findings and policy-making. How best to advocate the critical evaluation of all information sources, and the delegation of the specific task to search for research findings, as suggested by the policy-makers, remains unresolved. Different approaches to analyzing these issues will be important for future research. The present study analyzed the utilization of research at one stage in the policy-making process – the selection of specific medicines for an EML. The types of factors influencing the utilization of research findings likely differs in other levels of policy-making – for example the decision to have an EML in the first place. Getting an overall analysis of all levels of policy-formulation will be important for future research. Finally, it will be important to continue examining the effectiveness of strategies used to improve the uptake of research findings, such as case studies of collaborative research projects between researchers and policy-makers and analyses of the use of knowledge brokers in bridging these two communities.

## Conclusion

In order to determine the factors influencing policy makers' utilization of research findings in a developing country, this qualitative study examined health policy-makers' experiences in the selection and updating of Mali's EML. Due to the nature of the methods used, there are limitations to how far these factors can be generalized to other settings or other health policies. That said, many of the factors that emerged in this research have been found in similar studies in the literature. These factors support the issues related to the "two-communities theory" common in the literature, and highlight the importance of bridging these communities in order to improve the uptake of research findings.

Improving the transfer of research to policy will require effort on the part of policy-makers, the research community, and third parties. Policy-makers are likely to increase their utilization of research findings if their competency in research methods is improved and the importance they place on research findings in the policy-making process is increased. Providing these policy-makers with information they feel they can trust is also essential. Researchers can also improve the uptake of their research by making efforts to investigate policy-relevant issues. Increased collaboration between researchers and policy-makers and continued and improved technical support from various networks and multi-national organizations will put policy-makers in a better position to make more informed decisions so that the best health policies may be implemented in settings where the population potentially has the most to gain.

## Competing interests

DM works for the Direction of Pharmaceuticals and Medicines within the Ministry of Health in Mali and will be taking part in the next updating of the Malian EML.

## Authors' contributions

MA conceived of the study, participated in its design, collected the data, performed the analysis, and wrote the initial draft of the manuscript. AF participated in the conception and design of the study, offered technical support to the principle investigator during implementation, contributed to the analysis and helped prepare the draft manuscript. DM contributed to the acquisition of data, and made critical comments on the draft manuscript. All authors read and approved the final manuscript.

## Supplementary Material

Additional File 1 **Interview guide**. In-depth, semi-structured interview guide used in the study. Click here for file
