# Supplementary material for: Factors influencing the utilization of research findings by health policy-makers in a developing country: the selection of Mali's essential medicines
Source: Health Res Policy Syst. 2007 Mar 5;5:2. doi: 10.1186/1478-4505-5-2 (PMC1820594; doi:10.1186/1478-4505-5-2)
Supplement: Additional File 1 — Interview guide. In-depth, semi-structured interview guide used in the study. [file 1478-4505-5-2-S1.doc]

# **Interview guide**

# **(Actual interview conducted in French)**

Section A: Context/introduction

1. Confirm the role of the interviewee in his/her decision-making status for the selection of Mali’s essential medicines list (EML). To what extent? How many years?

***Section B: The decision making process***

1. Could you describe the process of selecting the medicines for the national list?
2. According to you, what is the purpose of an essential medicines list?
3. What do you think are the ***most*** important criteria to consider when selecting a drug for the EML? [Probe: safety, access, price, efficacy, pattern of prevalent diseases, current use in the country, single compound, generic available, availability on the international market]

***Section C: The informants; information mapping***

1. From whom do you take advice during the process of updating the essential medicines list?
2. Do you take advice from “non-experts” whose values you greatly trust?
3. Which other individuals, groups, or organisations are important? [Probe: ministers; legislature; officials; networks; professional groups; advocacy groups; academics/researchers; specific research centres; international organisations; industry; NGOs; political parties; religious leaders; mass media; the public]
4. Is there a situation when you would want to select a medicine that ***is not*** on the WHO essential medicines list? Example?
5. Is there a situation when you ***would not*** want to select a medicine that ***is*** on the WHO essential medicines list? Example?
6. To which ways of communicating or discussing the information with informants were you most receptive, or found most useful.

# Section D: Importance of scientific research for making decisions

1. What type of information do you consider most important when considering the addition or removal of a medicine to/from the list?
2. How important do you consider scientific research when making decisions about which medicines to select for the National EML? [Probe: relative importance of experience compared to research]
3. How well equipped was the selections committee to absorb research findings? [Probes: levels of training that members had in research methods; the use of policy analysis; the willingness to participate in official committees of policy-makers and scientists; and the degree of contact built up with researchers].

# Section E: Specific utilization of research

# Can you think of an example of a particularly difficult decision that was made? (Probes: WHO vs Mali list; old list vs new list; traditional medicines)

1. Were you able to use research findings to help make that decision? If so, from which source? From whom? [Probes: national or international research; systematic reviews; meta-analyses; Cochrane; national or international scientific literature; research reports; briefs of research findings produced by researchers; direct communication with individual researchers; attendance at seminars where research findings were presented; liaison with research centres; reports from official policy/science committees; briefs from research brokers/promoters/translators; briefs from policy advisers or officials; networks consisting of interest groups and other stakeholders; mass media; and dialogue with international agencies. Also Probe: informants mentioned in Section C]
2. Were there specific features of this research that made it useful? [Probe: type of information it provided, quality, timeliness]
3. Can you think of an example where more research information was needed?
4. Can you think of an example where the research presented was not useful to help you make your decision?
5. Were there reasons why research did not influence the decision to the extent that it could have done?
6. What factors do you believe could make policy-makers more receptive to research?

***Section F: Research as support***

1. Were the research findings useful in supporting the decisions, to help communicate the policy or to generate support for it in terms of financial resources, political commitment, public opinion?
2. Were the findings drawn upon in any speech, article, interview, report etc given to anyone at a higher level to support the decisions?

# Section G: Unique to the list: traditional medicines - if not discussed above

1. Why did you decide to look into traditional medicines?
2. Were there any particular research findings that were used for deciding to choose these 7 medicines? Please give examples.
3. Who were the informants that led to the addition of these medicines to the list?
4. Why are these medicines in a separate category of their own instead of being placed in their respective treatment categories?
5. Why are the improved traditional medicines on the essential medicines list but not in the therapeutic guidelines?

# Section H: Conclusion

1. Do you have anything else you might like to add, specifically related to the main question here: what are the factors influencing policy-makers’ utilization of research findings?

# Section I: Document checklist

1. Terms of reference for committee or guidelines for the selection proceedings
2. Minutes from meetings with the selections committees
3. Submissions to the committee
4. Any other material used by selections committee
